# Decoding emotional resilience in aging: unveiling the interplay between daily functioning and emotional health

**DOI:** 10.3389/fpubh.2024.1391033

**Published:** 2024-04-17

**Authors:** Minhua Guo, Songyang Xu, Xiaofang He, Jiawei He, Hui Yang, Lin Zhang

**Affiliations:** ^1^School of Integrated Chinese and Western Medicine, Hunan University of Chinese Medicine, Changsha, Hunan, China; ^2^Key Laboratory of Hunan Province for Integrated Traditional Chinese and Western Medicine on Prevention and Treatment of Cardio-Cerebral Diseases, College of Integrated Traditional Chinese and Western Medicine, Hunan University of Chinese Medicine, Changsha, Hunan, China; ^3^School of Mechatronics and Energy Engineering, NingboTech University, Ningbo, China; ^4^Nursing Department, Guizhou Provincial People's Hospital, Guiyang, Guizhou, China; ^5^Department of Neurology, The Second Affiliated Hospital of Guizhou University of Chinese Medicine, Guiyang, Guizhou, China

**Keywords:** activities of daily living (ADL), emotional problems (EPs), machine learning algorithm, the China health and retirement longitudinal survey (CHARLS), Barthel index (BI), support vector machine (SVM), decision tree (DT), logistic regression (LR)

## Abstract

**Background:**

EPs pose significant challenges to individual health and quality of life, attracting attention in public health as a risk factor for diminished quality of life and healthy life expectancy in middle-aged and older adult populations. Therefore, in the context of global aging, meticulous exploration of the factors behind emotional issues becomes paramount. Whether ADL can serve as a potential marker for EPs remains unclear. This study aims to provide new evidence for ADL as an early predictor of EPs through statistical analysis and validation using machine learning algorithms.

**Methods:**

Data from the 2018 China Health and Retirement Longitudinal Study (CHARLS) national baseline survey, comprising 9,766 samples aged 45 and above, were utilized. ADL was assessed using the BI, while the presence of EPs was evaluated based on the record of “Diagnosed with Emotional Problems by a Doctor” in CHARLS data. Statistical analyses including independent samples *t*-test, chi-square test, Pearson correlation analysis, and multiple linear regression were conducted using SPSS 25.0. Machine learning algorithms, including Support Vector Machine (SVM), Decision Tree (DT), and Logistic Regression (LR), were implemented using Python 3.10.2.

**Results:**

Population demographic analysis revealed a significantly lower average BI score of 65.044 in the “Diagnosed with Emotional Problems by a Doctor” group compared to 85.128 in the “Not diagnosed with Emotional Problems by a Doctor” group. Pearson correlation analysis indicated a significant negative correlation between ADL and EPs (*r* = −0.165, *p* < 0.001). Iterative analysis using stratified multiple linear regression across three different models demonstrated the persistent statistical significance of the negative correlation between ADL and EPs (B = −0.002, *β* = −0.186, *t* = −16.476, 95% CI = −0.002, −0.001, *p* = 0.000), confirming its stability. Machine learning algorithms validated our findings from statistical analysis, confirming the predictive accuracy of ADL for EPs. The area under the curve (AUC) for the three models were SVM-AUC = 0.700, DT-AUC = 0.742, and LR-AUC = 0.711. In experiments using other covariates and other covariates + BI, the overall prediction level of machine learning algorithms improved after adding BI, emphasizing the positive effect of ADL on EPs prediction.

**Conclusion:**

This study, employing various statistical methods, identified a negative correlation between ADL and EPs, with machine learning algorithms confirming this finding. Impaired ADL increases susceptibility to EPs.

## 1 Introduction

As the world’s population continues to age, projections indicate that the number of individuals aged 60 and above in developing nations will surge from 900 million to two billion between 2015 and 2050 (1). Among the older adult population, the activities of daily living (ADL) and emotional problems (EPs) have received widespread attention in the fields of medicine and public health research (2–6). EPs encompass a range of disorders such as panic disorder, generalized anxiety disorder, social phobia, specific phobia, obsessive-compulsive disorder, post-traumatic stress disorder, and depression (7). These EPs pose significant challenges to individual health and quality of life (8). Research has found that the overall prevalence of depression among older adults globally is 28.4% (9), and depression may accelerate the cellular aging process (10). Symptoms of depression, anxiety, and other emotional issues also manifest in the preclinical stages of Alzheimer’s disease (AD) (11). EPs greatly impact the health and lifespan of the older adult. Therefore, in the context of global aging, it is particularly important to meticulously explore the factors behind emotional issues.

EPs, characterized primarily by emotional disturbances such as depression, anxiety, mania, and feelings of loneliness, may also entail impulsive behavior, disruptions in sleep and diet, and even suicidal or self-harming thoughts (12). For middle-aged and older adult individuals, the pressures of work and family, or difficulties adjusting to life after retirement, coupled with disparities in social interaction and attention, can lead to emotional disruptions, resulting in a spectrum of EPs (13). In the prodromal stages of AD, which often coincide with gradual and insidious impairments in ADL, individuals may experience EPs, manifesting as depression or anxiety, yet these symptoms often go unnoticed, leading to exacerbation in later stages of AD (14, 15). During the recovery and sequelae phases of stroke, EPs are often present (16), with patients frequently experiencing difficulty in emotional regulation, thereby impacting stroke rehabilitation (17, 18). ADL encompasses a range of pertinent issues such as personal self-care, proficiency in functional tasks, and the ability to perform activities, serving as a cornerstone of an individual’s quality of life (19). Age-related impairments in ADL may render middle-aged and older adult individuals unable to accept declines in physical function and personal capabilities, thereby triggering EPs (20). Encouraging patients to improve their ADL is a crucial measure in the rehabilitation of EPs, as enhancing ADL can aid patients in returning to normal life and stabilizing their emotions (21). In stroke rehabilitation, as patients’ physical abilities improve, activities such as independent eating, walking, and climbing stairs can alleviate EPs resulting from stroke sequelae (22, 23).

Previous studies have hinted at the potential correlation between ADL and EPs. To elucidate this correlation further, the present study collected data from the China Health and Retirement Longitudinal Survey (CHARLS) in 2018 and employed various statistical methods for analysis, revealing a significant correlation between ADL and EPs. Additionally, three machine learning algorithms—Support Vector Machine (SVM), Decision Tree (DT), and Logistic Regression (LR)—were employed to validate our findings. The primary objective of this study is to elucidate the association between ADL and EPs, enhance the awareness of ADL among middle-aged and older adult individuals, and introduce novel avenues for non-pharmacological rehabilitation therapy for emotional issues in this demographic group.

## 2 Methods

### 2.1 Study population

This study collected data from the China Health and Retirement Longitudinal Study (CHARLS) baseline dataset, which is operated by the National School of Development at Peking University.^1^ CHARLS has established a high-quality open-access database encompassing various details regarding individuals, families, health status, and socio-economic aspects, including “Health Status and Functioning,” “Cognition,” “Work Retirement,” and “Family Information.” The study cohort comprised individuals aged 45 and above randomly selected from 150 counties or districts and 450 villages or urban areas across 28 provinces, representing the middle-aged and older adult population in China (24). Utilizing the 2018 CHARLS dataset, this study encompassed a total of 20,813 samples. Participants under 45 years old were excluded from the analysis, along with those with insufficient demographic or health data, resulting in a final sample size of 9,766 individuals.

### 2.2 Assessment of emotional problems

The assessment was conducted based on the records of emotional problems data “Diagnosed with Emotional Problems by a Doctor” in CHARLS. The samples were divided into two cohorts: those affirmed as “Diagnosed with Emotional Problems by a Doctor” and those negated as “Not diagnosed with Emotional Problems by a Doctor.”

### 2.3 Assessment of ADL

The Barthel Index (BI) is a recognized method used to assess the level of ADL. In this study, the BI was employed to evaluate the ADL of all samples. The BI categorizes daily activities into 10 independent elements, including “Feeding,” “Bathing,” “Grooming,” “Dressing,” “Bowel Control,” “Bladder Control,” “Toilet Use,” “Transfers,” “Mobility,” and “Stairs,” each element being assessed with a score (Table 1), reflecting the individual’s ability and proficiency in completing the respective tasks. By assigning scores to each element and summarizing these scores, the BI is derived, ranging from 0 to 100. A higher BI score indicates greater autonomy and proficiency in performing daily life activities (6, 25–27).

**Table 1:** Activities of daily living scale.

Activities of daily living	Independent	Partially independent	Moderately dependent	Totally dependent
Feeding	10	5	0	0
Bathing	5	0	0	0
Grooming	5	0	0	0
Dressing	10	5	0	0
Transfers	15	10	5	0
Mobility	15	10	5	0
Stairs	10	5	0	0
Toilet Use	10	5	0	0
Bowel Control	10	5	0	0
Bladder Control	10	5	0	0

### 2.4 Assessment of covariates

The covariates in this study were derived from the CHARLS dataset, encompassing variables such as age, gender (male, female), residence (Central of City/Town, Urban–Rural Integration Zone, Rural, Special Zone), education (ranging from No Formal Education (Illiterate) to Doctoral Degree/Ph.D.), smoking status (Still Smoke, Quit or No, Never Smoked), drinking status (Drink more than once a month, Drink but less than once a month, None), as well as hypertension, diabetes, and dyslipidemia. For age, middle-aged individuals were defined as those aged 45 to below 60 years, while older adult individuals were defined as those aged 60 years and above. Regarding hypertension, as per the 2010 Chinese Hypertension Guidelines, hypertension was diagnosed based on an average systolic blood pressure ≥ 140mmHg, and/or an average diastolic blood pressure ≥ 90mmHg, and/or self-reported use of antihypertensive medications within the past 2 weeks (28). Diabetes diagnosis criteria included a fasting blood glucose level ≥ 7.0 mmol/L or current treatment with antidiabetic medication. Dyslipidemia diagnosis criteria, following the 2016 Chinese Adult Dyslipidemia Guidelines, included total cholesterol level ≥ 240 mg/dL, high-density lipoprotein cholesterol level < 40 mg/dL, low-density lipoprotein cholesterol level > 160 mg/dL, or triglyceride level ≥ 200 mg/dL (29).

### 2.5 Statistical analysis

In the demographic characteristics analysis, continuous variables such as age and BI were summarized using mean and standard deviation (SD), while categorical variables like gender, residence, and education were represented as counts and percentages. To examine demographic characteristics, independent sample *t*-tests were employed for continuous variables to discern differences, while chi-square tests were utilized for categorical variables. Correlation analysis involved Pearson correlation tests to assess the relationship between independent variables, covariates, and dependent variables. Subsequently, a comprehensive evaluation of the relationship between ADL and EPs was conducted through hierarchical multiple linear regression analysis. This regression underwent three iterations with distinct models, incorporating dummy variables as references in each variable. Model 1 included three variables: age, gender, and ADL. Model 2 extended Model 1 by adding residence, education, smoking status, and drinking status, resulting in a total of seven variables. Model 3 further expanded upon Model 2 with the inclusion of hypertension, diabetes, and dyslipidemia, encompassing a total of 10 variables.

### 2.6 Machine learning algorithm

#### 2.6.1 Parameter setup

The machine learning algorithms were implemented using the PyCharm 2023.1.2 integrated development environment and executed on a computer running the Windows 11 operating system. The software development process was conducted within the specified Anaconda development environment, utilizing Python version 3.10.2 for coding and analysis. A random seed number of 42 was set and maintained consistently across the entire process to ensure the reproducibility and consistency of results. The original dataset was partitioned into two distinct subsets: a training set and a testing set. The training set comprised 70% of the original data, while the remaining 30% was allocated to the testing set. The data was randomly shuffled, and the model’s performance was evaluated by examining the classification results generated from the testing dataset.

#### 2.6.2 Experimental models

##### 2.6.2.1 Support vector machine

The SVM stands out as a prominent supervised learning technique within the realm of machine learning, classified as a generalized linear classifier. Its core objective revolves around accurately classifying data points by discerning the maximum-margin hyperplane, which serves as the decision boundary for the training dataset. One of SVM’s distinguishing features is its adaptability in tackling nonlinear classification tasks, facilitated by the utilization of kernel functions. This capability underscores SVM’s pivotal role in kernel-based learning methodologies. Notably, SVM demonstrates remarkable efficacy in addressing high-dimensional problems, including those characterized by expansive feature spaces, and exhibits prowess in navigating intricate relationships among nonlinear features (30, 31).

##### 2.6.2.2 Decision tree learning

DT, a widely used supervised technique in statistics, data mining, and machine learning, offers versatile classification and regression capabilities. These trees, fashioned as predictive models, derive insights from observational data. In classification, they serve as tree-based models for discrete target variable values, with terminal nodes denoting class labels and branches delineating feature combinations leading to these labels. In regression, decision trees adeptly handle continuous target variable values, typically real numbers. Their adaptability spans various data types, encompassing categorical sequences, thereby augmenting their versatility across diverse analytical domains. Known for their simplicity and interpretability, decision trees are valuable tools for transparent decision-making processes and are particularly useful in decision analysis and data mining applications, where they provide descriptive insights and aid in decision-making (32–34).

##### 2.6.2.3 Logistic regression

LR (Equation 1), a widely embraced methodology in supervised learning within machine learning, is distinct from linear regression as it primarily tackles classification problems, even in multi-classification scenarios. During training, the model learns from a dataset comprising multiple groups, known as the training set, absorbing patterns crucial for classification decisions. After training, the model applies this acquired knowledge to classify one or more datasets, referred to as the test set, each characterized by various indicators contributing to the decision-making process. Logistic regression’s adaptability renders it indispensable in supervised learning, playing a foundational role in machine learning methodologies across scientific and medical domains (35–37).

(1) $$sigmoid=\frac{1}{1+e^{-z}}$$

Equation 1: Logistic regression.

#### 2.6.3 Evaluating indicator

This study utilized accuracy, precision, recall, and F1-score as the selected evaluation metrics (38, 39). Accuracy assesses the proportion of correctly predicted labels to the total labels (Equation 2). Precision evaluates the proportion of true positive predictions among all positive predictions (Equation 3). Recall gauges the proportion of true positive predictions among all actual positive instances (Equation 4). F1-score reflects the robustness of the model, simultaneously considering precision and recall. A higher F1 value indicates better model performance (Equation 5).

(2) $$Accuracy=\frac{TP+TN}{TP+FP+TN+FN}$$

Equation 2: Accuracy.

(3) $$Precision=\frac{TP}{TP+FP}$$

Equation 3: Precision.

(4) $$Recall=\frac{TP}{TP+FN}$$

Equation 4: Recall.

(5) $$F1=\frac{2TP}{2TP+FN+FP}=\frac{2*Precision*Recall}{Precision+Recall}$$

Equation 5: F1-score (TP, True Positive; FP, False Positive; FN, False Negative; TN, True Negative).

## 3 Results

### 3.1 Characteristics of samples

The study rigorously excluded ineligible subjects, namely those aged 45 and below, as well as individuals lacking adequate demographic or health data. Ultimately, the cohort comprised 9,766 participants, among whom 3,904 were male (39.98%) and 5862 were female (60.02%). Table 2 encapsulates the demographic characteristics of the study cohort, wherein 9539 individuals (97.68%) remained undiagnosed with EPs by medical professionals, while 227 individuals (2.32%) received EP diagnoses. Remarkably, those diagnosed with EPs demonstrated substantially lower average BI scores (65.044, standard deviation = 30.6421) compared to their undiagnosed counterparts (85.128, standard deviation = 17.6386), indicating a significant disparity (*p* < 0.001, *t* = 16.573) in ADL between the diagnosed and undiagnosed EP cohorts. Furthermore, significant differences (*p* < 0.001) were discerned between individuals diagnosed with EPs and those without in terms of gender (*p* = 0.015), smoking status (*p* = 0.005), alcohol consumption (*p* < 0.001), hypertension (*p* = 0.001), and dyslipidemia (*p* = 0.011). Analysis of the demographic features of the sample underscores the influence of ADL, gender, education, smoking and alcohol habits, as well as the presence of hypertension or dyslipidemia on the prevalence of EPs.

**Table 2:** Demographic characteristics of middle-aged and older adults Chinese with and without emotional problems by doctor.

Variables	Not diagnosed with emotional problems by a doctor	Diagnosed with emotional problems by a doctor	*p*-value	*t*/χ^2^
No. subjects (%)	9539(97.68)	227(2.32)		
Age, year			0.157	1.415
	65.14267743	66.1277533		
SD	10.36763582	10.15416304		
Gender, n (%)			0.015	5.918
Male	3831(40.16)	73(32.16)		
Female	5708(59.84)	154(67.84)		
Residence, n (%)			0.105	6.150
Central of City/Town	1614(16.92)	35(15.42)		
Urban–Rural Integration Zone	628(6.58)	24(10.57)		
Rural	7274(76.26)	167(73.57)		
Special Zone	23(0.24)	1(0.44)		
Education, n (%)				
No Formal Education (Illiterate)	2083(21.84)	51(22.47)	<0.001	44.91
Did not Finish Primary School	1839(19.28)	39(17.18)		
Sishu/Home School	18(0.19)	1(0.44)		
Elementary School	2016(21.13)	50(22.03)		
Middle School	2172(22.77)	53(23.35)		
High School	856(8.97)	19(8.37)		
Vocational School	282(2.96)	8(3.52)		
Two-/Three-Year College/Associate Degree	157(1.65)	4(1.76)		
Four-Year College/Bachelor’s Degree	105(1.10)	1(0.44)		
Master’s Degree	11(0.12)	0(0)		
Doctoral Degree/Ph.D.	0(0)	1(0.44)		
Smoking status, n (%)			0.005	10.713
Still Smoke	2131(22.34)	30(13.22)		
Quit or No	7293(76.45)	194(85.46)		
Never Smoked	115(1.21)	3(1.32)		
Drinking status, n (%)			<0.001	21.67
Drink more than once a month	1895(19.87)	23(10.13)		
Drink less than once a month	630(6.60)	6(2.64)		
None	7014(73.53)	198(87.22)		
Hypertension, n (%)				
Yes	1799(18.86)	62(27.31)	0.001	10.272
No	7740(81.14)	165(72.69)		
Diabetes, n (%)				
Yes	650(6.81)	18(7.93)	0.511	0.433
No	8889(93.19)	209(92.07)		
Dyslipidemia, n (%)				
Yes	1256(13.17)	43(18.94)	0.011	6.414
No	8283(86.83)	184(81.06)		
Activity of daily living (The Barthel Index)			<0.001	−16.573
	85.128	65.044		
SD	17.6386	30.6421		

### 3.2 The correlation between variable and EPs

We employed Pearson correlation analysis to explore the relationship between EPs and both independent and covariate variables (Table 3). The results reveal a significant negative correlation between ADL and EPs (*r* = −0.165, *p* < 0.001), indicating an increased susceptibility to EPs among individuals with impaired ADL. Additionally, a slight yet significant positive correlation was observed between gender and EPs (*r* = 0.025, *p* = 0.015), suggesting a higher likelihood of females being diagnosed with EPs. Regarding smoking status, a significant positive correlation with EPs was found (*r* = 0.032, *p* = 0.002), implying a higher susceptibility to EPs among non-smokers. Similarly, a significant positive correlation was observed between alcohol consumption and EPs (*r* = 0.044, *p* < 0.001), indicating that individuals with lower alcohol consumption frequency are more likely to be diagnosed with EPs. Furthermore, hypertension (*r* = 0.032, *p* = 0.001) and dyslipidemia (*r* = 0.026, *p* = 0.011) exhibited significant positive correlations with EPs, suggesting a greater likelihood of individuals with hypertension or dyslipidemia being diagnosed with EPs.

**Table 3:** Correlation between variable and emotional problems.

Variables	Emotional problems	
	*r*	*p-*value
Age	0.014	0.157
Gender	0.025	0.015
Residence	−0.002	0.878
Education	0.002	0.842
Smoking status	0.032	0.002
Drinking status	0.044	<0.001
Hypertension	0.032	0.001
Diabetes	0.007	0.511
Dyslipidemia	0.026	0.011
Activity of daily living(The Barthel Index)	−0.165	<0.001

Emotional problems: 0 = “Not diagnosed with Emotional Problems by a Doctor,” 1 = “Diagnosed with Emotional Problems by a Doctor”; Gender:1 = “Male,”2 = “Female”;Residence:1 = “Central of City/Town “,2 = “Urban–Rural Integration Zone,”3 = “Rural,”4 = “Special Zone”;Education:1 = “No Formal Education (Illiterate),”2 = “Did not Finish Primary School,”3 = “Sishu/Home School,”4 = “Elementary School,”5 = “Middle School,”6 = “High School,”7 = “Vocational School,”8 = “Two-/Three-Year College/Associate Degree,”9 = “Four-Year College/Bachelor’s Degree,”10 = “Master’s Degree,”11 = “Doctoral Degree/Ph.D.”; Smoking status:1 = “Still Smoke,”2 = “Quit or No,”3 = “Never Smoked”; Drinking status:1 = “Drink More than Once a Month,”2 = “Drink But Less than Once a Month,” 3 = “None”; Hypertension/Diabetes/Dyslipidemia:0 = “No,”1 = “Yes.”

### 3.3 Associations between ADL and EPs

To delve deeper into the correlation between EPs and ADL, we employed hierarchical multiple linear regression to systematically investigate the association between the independent variables (ADL, age, gender, residence, smoking status, etc.) and the dependent variable EPs across three distinct models (Model 1, Model 2, Model 3; Table 4).

**Table 4:** Associations between ADL and emotional problems in middle-aged and older adults Chinese.

Model	R	R square	F	*p-*value	Variables		B	*β*	*t*	95%CI		*p*-value
Model 1	0.170	0.029	96.764	<0.001		ADL	−0.001	−0.175	−16.777	−0.002	−0.001	0.000
						Age	−0.001	−0.036	−3.446	−0.001	0.000	0.001
					Gender	Male	−0.005	−0.015	−1.489	−0.011	0.001	0.137
						Female(Ref.)						
Model 2	0.187	0.035	17.752	<0.001		ADL	−0.001	−0.174	−16.528	−0.002	−0.001	0.000
						Age	−0.001	−0.037	−3.553	−0.001	0.000	0.000
					Gender	Male	0.000	0.000	−0.013	−0.007	0.007	0.990
						Female(Ref.)						
					Residence	Central of City/Town	−0.018	−0.044	−0.576	−0.077	0.042	0.564
						Urban–Rural Integration Zone	−0.004	−0.007	−0.138	−0.065	0.056	0.890
						Rural	−0.023	−0.065	−0.758	−0.082	0.036	0.448
						Special Zone(Ref.)						
					Education	No Formal Education (Illiterate)	−0.966	−2.648	−6.514	−1.256	−0.675	0.000
						Did not Finish Primary School	−0.969	−2.534	−6.536	−1.259	−0.678	0.000
						Sishu/Home School	−0.936	−0.274	−6.157	−1.234	−0.638	0.000
						Elementary School	−0.965	−2.616	−6.511	−1.256	−0.675	0.000
						Middle School	−0.966	−2.688	−6.515	−1.256	−0.675	0.000
						High School	−0.969	−1.836	−6.533	−1.259	−0.678	0.000
						Vocational School	−0.963	−1.085	−6.485	−1.254	−0.672	0.000
						Two-/Three-Year College/Associate Degree	−0.969	−0.819	−6.521	−1.261	−0.678	0.000
						Four-Year College/Bachelor’s Degree	−0.978	−0.672	−6.565	−1.269	−0.686	0.000
						Master’s Degree	−0.981	−0.218	−6.338	−1.285	−0.678	0.000
						Doctoral Degree/Ph.D.(Ref.)						
					Smoking status	Still Smoke	−0.006	−0.016	−0.420	−0.033	0.022	0.674
						Quit or No	−0.002	−0.005	−0.131	−0.029	0.025	0.895
						Never Smoked(Ref.)						
					Drinking status	Drink more than once a month	−0.007	−0.020	−1.759	−0.016	0.001	0.079
						Drink less than once a month	−0.013	−0.021	−2.033	−0.025	0.000	0.042
						No(Ref.)						
Model 3	0.190	0.036	15.870	<0.001		ADL	−0.002	−0.186	−16.476	−0.002	−0.001	0.000
						Age	−0.001	−0.037	−3.535	−0.001	0.000	0.000
					Gender	Male	0.000	0.000	0.041	−0.007	0.008	0.968
						Female(Ref.)						
					Residence	Central of City/Town	−0.017	−0.043	−0.567	−0.077	0.042	0.571
						Urban–Rural Integration Zone	−0.004	−0.006	−0.123	−0.064	0.057	0.902
						Rural	−0.023	−0.065	−0.761	−0.082	0.036	0.446
						Special Zone(Ref.)						
					Education	No Formal Education (Illiterate)	−0.963	−2.641	−6.499	−1.254	−0.673	0.000
						Did not Finish Primary School	−0.966	−2.528	−6.521	−1.257	−0.676	0.000
						Sishu/Home School	−0.932	−0.273	−6.130	−1.230	−0.634	0.000
						Elementary School	−0.963	−2.609	−6.497	−1.253	−0.672	0.000
						Middle School	−0.963	−2.682	−6.501	−1.254	−0.673	0.000
						High School	−0.966	−1.831	−6.517	−1.257	−0.675	0.000
						Vocational School	−0.960	−1.082	−6.471	−1.251	−0.669	0.000
						Two-/Three-Year College/Associate Degree	−0.967	−0.817	−6.506	−1.258	−0.676	0.000
						Four-Year College/Bachelor’s Degree	−0.975	−0.671	−6.553	−1.267	−0.684	0.000
						Master’s Degree	−0.977	−0.217	−6.310	−1.280	−0.673	0.000
						Doctoral Degree/Ph.D.(Ref.)						
					Smoking status	Still Smoke	−0.007	−0.019	−0.488	−0.034	0.021	0.626
						Quit or No	−0.003	−0.008	−0.197	−0.030	0.024	0.844
						Never Smoked(Ref.)						
					Drinking status	Drink more than once a month	−0.008	−0.020	−1.813	−0.016	0.001	0.070
						Drink less than once a month	−0.012	−0.020	−2.016	−0.025	0.000	0.044
						No(Ref.)						
					Hypertension	Yes	−0.012	−0.030	−2.781	−0.020	−0.003	0.005
						No(Ref.)						
					Diabetes	Yes	−0.007	−0.011	−1.104	−0.019	0.005	0.270
						No(Ref.)						
					Dyslipidemia	Yes	0.000	0.000	0.026	−0.009	0.009	0.979
						No(Ref.)						

In Model 1, we included ADL, age, and gender (*R* = 0.170, *R^2^* = 0.029, *F* = 96.764, *p* < 0.001). We observed a significant negative correlation between ADL and EPs (*B* = −0.001, *β* = −0.175, *t* = −16.777, 95% CI = −0.002, −0.001, *p* = 0.000), underscoring the heightened susceptibility to EPs among individuals with impaired daily living abilities. In Model 2, additional covariates were incorporated (residence, education, smoking, and drinking status), where ADL continued to exhibit a pronounced negative correlation with EPs (*B* = −0.001, *β* = −0.174, *t* = −16.528, 95% CI = −0.002, −0.001, *p* = 0.000), maintaining statistical significance. In the final iteration, Model 3 introduced hypertension, dyslipidemia, and diabetes, reaffirming the negative correlation between ADL and EPs (*B* = −0.002, *β* = −0.186, *t* = −16.476, 95% CI = −0.002, −0.001, *p* = 0.000), with an overall significant fit (*R* = 0.190, *R^2^* = 0.036, *F* = 15.870, *p* < 0.001).

All three models demonstrated statistically significant associations between the independent and dependent variables (*p* < 0.001). The R value increased gradually from 0.170 in Model 1 to 0.190 in Model 3, indicating an enhanced ability to explain the variability of outcomes. Across all models, despite adjustments for covariates, our study consistently emphasized the significant association between declining ADL and increased susceptibility to EPs, while underscoring the robustness of this relationship.

In our investigation, hypertension emerged as a consistent factor exhibiting correlation with EPs across all analyses, suggesting its potential role as a significant confounding factor in this study. To mitigate this confounding effect, we partitioned the entire sample into two cohorts: a hypertension group (Table 5) and a non-hypertension group (Table 6). Through this stratification, we aimed to delve into the relationship between ADL and EPs in detail.

**Table 5:** Associations between ADL and EPs in the hypertension group.

Model	R	R square	F	*p*-value	Variables		B	*β*	*t*	95%CI		P-value
Model 1	0.134	0.018	11.301	<0.001		ADL	−0.001	−0.141	−5.750	−0.001	−0.001	0.000
						Age	−0.001	−0.053	−2.171	−0.002	0.000	0.030
					Gender	Male	−0.005	−0.014	−0.591	−0.021	0.012	0.554
						Female(Ref.)						
Model 2	0.156	0.024	2.430	<0.001		ADL	−0.001	−0.130	−5.141	−0.001	−0.001	0.000
						Age	−0.001	−0.057	−2.293	−0.002	0.000	0.022
					Gender	Male	0.006	0.017	0.628	−0.013	0.026	0.530
						Female(Ref.)						
					Residence	Central of City/Town	0.027	0.055	0.297	−0.150	0.204	0.767
						Urban–Rural Integration Zone	0.050	0.071	0.544	−0.129	0.228	0.586
						Rural	0.032	0.075	0.353	−0.144	0.208	0.724
						Special Zone(Ref.)						
					Education	No Formal Education (Illiterate)	0.020	0.046	0.225	−0.156	0.196	0.822
						Did not Finish Primary School	0.025	0.055	0.274	−0.152	0.201	0.784
						Sishu/Home School	−0.004	−0.001	−0.039	−0.230	0.222	0.969
						Elementary School	0.024	0.054	0.263	−0.153	0.200	0.793
						Middle School	0.034	0.078	0.381	−0.142	0.210	0.703
						High School	0.019	0.031	0.206	−0.159	0.196	0.837
						Vocational School	0.028	0.026	0.300	−0.154	0.209	0.764
						Two-/Three-Year College/Associate Degree	0.047	0.035	0.494	−0.139	0.232	0.622
						Four-Year College/Bachelor’s Degree	−0.006	−0.003	−0.061	−0.202	0.190	0.951
						Doctoral Degree/Ph.D.(Ref.)						
					Smoking status	Still Smoke	0.032	0.070	0.943	−0.034	0.097	0.346
						Quit or No	0.042	0.097	1.285	−0.022	0.106	0.199
						Never Smoked(Ref.)						
					Drinking status	Drink more than once a month	−0.022	−0.045	−1.742	−0.046	0.003	0.082
						Drink less than once a month	−0.031	−0.041	−1.723	−0.066	0.004	0.085
						No(Ref.)						
Model 3	0.163	0.026	2.383	<0.001		ADL	−0.001	−0.123	−4.784	−0.001	−0.001	0.000
						Age	−0.001	−0.052	−2.085	−0.002	0.000	0.037
					Gender	Male	0.005	0.014	0.504	−0.015	0.025	0.614
						Female(Ref.)						
					Residence	Central of City/Town	0.023	0.047	0.256	−0.154	0.200	0.798
						Urban–Rural Integration Zone	0.045	0.064	0.494	−0.134	0.223	0.622
						Rural	0.029	0.070	0.328	−0.147	0.205	0.743
						Special Zone(Ref.)						
					Education	No Formal Education (Illiterate)	0.018	0.041	0.201	−0.158	0.194	0.841
						Did not Finish Primary School	0.022	0.048	0.242	−0.154	0.198	0.808
						Sishu/Home School	−0.007	−0.002	−0.065	−0.233	0.219	0.948
						Elementary School	0.021	0.049	0.238	−0.155	0.197	0.812
						Middle School	0.032	0.072	0.352	−0.144	0.208	0.725
						High School	0.016	0.027	0.178	−0.161	0.193	0.859
						Vocational School	0.027	0.026	0.292	−0.154	0.208	0.770
						Two-/Three-Year College/Associate Degree	0.043	0.032	0.460	−0.142	0.229	0.646
						Four-Year College/Bachelor’s Degree	−0.010	−0.005	−0.101	−0.206	0.186	0.920
						Master’s Degree(Ref.)						
					Smoking status	Still Smoke	0.033	0.073	0.978	−0.033	0.099	0.328
						Quit or No	0.042	0.097	1.289	−0.022	0.107	0.198
						Never Smoked(Ref.)						
					Drinking status	Drink more than once a month	−0.022	−0.045	−1.762	−0.046	0.002	0.078
						Drink less than once a month	−0.030	−0.040	−1.704	−0.065	0.005	0.089
						No(Ref.)						
					Diabetes	Yes	−0.015	−0.028	−1.195	−0.040	0.010	0.232
						No(Ref.)						
					Dyslipidemia	Yes	0.017	0.043	1.766	−0.002	0.036	0.078
						No(Ref.)						

**Table 6:** Associations between ADL and EPs in the non-hypertension group.

Model	R	R square	F	*p*-value	Variables		B	*β*	*t*	95%CI		*p*-value
Model 1	0.193	0.037	102.236	<0.001		ADL	−0.002	−0.198	−17.273	−0.002	−0.002	0.000
						Age	0.000	−0.033	−2.877	−0.001	0.000	0.004
					Gender	Male	−0.004	−0.014	−1.294	−0.011	0.002	0.196
						Female(Ref.)						
Model 2	0.202	0.041	17.672	<0.001		ADL	−0.002	−0.201	−17.403	−0.002	−0.002	0.000
						Age	−0.001	−0.036	−3.149	−0.001	0.000	0.002
					Gender	Male	−0.001	−0.004	−0.278	−0.009	0.007	0.781
						Female(Ref.)						
					Residence	Central of City/Town	−0.028	−0.073	−0.875	−0.090	0.034	0.382
						Urban–Rural Integration Zone	−0.018	−0.031	−0.552	−0.080	0.045	0.581
						Rural	−0.037	−0.110	−1.176	−0.099	0.025	0.240
						Special Zone(Ref.)						
					Education	No Formal Education (Illiterate)	−0.107	−0.309	−2.148	−0.204	−0.009	0.032
						Did not Finish Primary School	−0.112	−0.308	−2.254	−0.209	−0.015	0.024
						Sishu/Home School	−0.047	−0.013	−0.751	−0.171	0.076	0.453
						Elementary School	−0.108	−0.307	−2.163	−0.205	−0.010	0.031
						Middle School	−0.111	−0.326	−2.224	−0.208	−0.013	0.026
						High School	−0.111	−0.219	−2.221	−0.209	−0.013	0.026
						Vocational School	−0.104	−0.124	−2.066	−0.203	−0.005	0.039
						Two-/Three-Year College/Associate Degree	−0.119	−0.105	−2.330	−0.219	−0.019	0.020
						Four-Year College/Bachelor’s Degree	−0.117	−0.087	−2.265	−0.219	−0.016	0.024
						Master’s Degree(Ref.)						
					Smoking status	Still Smoke	−0.022	−0.064	−1.418	−0.052	0.008	0.156
						Quit or No	−0.019	−0.057	−1.261	−0.049	0.011	0.207
						Never Smoked(Ref.)						
					Drinking status	Drink more than once a month	−0.005	−0.015	−1.166	−0.014	0.004	0.244
						Drink less than once a month	−0.009	−0.016	−1.413	−0.022	0.004	0.158
						No(Ref.)						
Model 3	0.203	0.041	16.081	<0.001		ADL	−0.002	−0.202	−17.450	−0.002	−0.002	0.000
						Age	−0.001	−0.037	−3.219	−0.001	0.000	0.001
					Gender	Male	−0.001	−0.004	−0.275	−0.009	0.007	0.783
						Female(Ref.)						
					Residence	Central of City/Town	−0.028	−0.073	−0.880	−0.090	0.034	0.379
						Urban–Rural Integration Zone	−0.018	−0.031	−0.553	−0.080	0.045	0.580
						Rural	−0.037	−0.111	−1.189	−0.099	0.024	0.235
						Special Zone(Ref.)						
					Education	No Formal Education (Illiterate)	−0.106	−0.306	−2.132	−0.203	−0.009	0.033
						Did not Finish Primary School	−0.111	−0.306	−2.237	−0.209	−0.014	0.025
						Sishu/Home School	−0.047	−0.013	−0.748	−0.171	0.076	0.455
						Elementary School	−0.107	−0.305	−2.149	−0.204	−0.009	0.032
						Middle School	−0.110	−0.324	−2.209	−0.207	−0.012	0.027
						High School	−0.110	−0.218	−2.208	−0.208	−0.012	0.027
						Vocational School	−0.104	−0.123	−2.053	−0.202	−0.005	0.040
						Two-/Three-Year College/Associate Degree	−0.118	−0.104	−2.312	−0.218	−0.018	0.021
						Four-Year College/Bachelor’s Degree	−0.116	−0.086	−2.250	−0.218	−0.015	0.024
						Master’s Degree(Ref.)						
					Smoking status	Still Smoke	−0.022	−0.065	−1.438	−0.052	0.008	0.151
						Quit or No	−0.019	−0.058	−1.278	−0.049	0.010	0.201
						Never Smoked(Ref.)						
					Drinking status	Drink more than once a month	−0.005	−0.015	−1.207	−0.014	0.003	0.227
						Drink less than once a month	−0.009	−0.016	−1.410	−0.022	0.004	0.158
						No(Ref.)						
					Diabetes	Yes	−0.003	−0.005	−0.467	−0.017	0.010	0.641
						No(Ref.)						
					Dyslipidemia	Yes	−0.007	−0.014	−1.255	−0.017	0.004	0.210
						No(Ref.)						

Within the hypertension group, iterative modeling across three iterations continued to reveal a statistically significant negative correlation between ADL and EPs (*B* = −0.001, *β* = −0.123, *t* = −4.784, 95% CI = −0.001, −0.001, *p* = 0.000). Similarly, within the non-hypertension group, this negative correlation persisted (*B* = −0.002, *β* = −0.202, *t* = −17.450, 95% CI = −0.002, −0.002, *p* = 0.000). These findings underscore that irrespective of hypertension status and adjustments for covariates, ADL continues to influence susceptibility to EPs (*p* < 0.001).

### 3.4 Machine learning algorithm confirm the link between ADL and EPs

Through statistical analysis, we have identified a significant negative correlation between ADL and EPs, indicating that individuals with impaired daily living abilities are more susceptible to EPs. Moreover, employing hierarchical multiple linear regression, adjusting for and iterating covariates, we have established the stability of the negative correlation between ADL and EPs. Consequently, we employed three machine learning algorithms—SVM, DT, and LR—to validate the association between the two.

**Figure 1:**
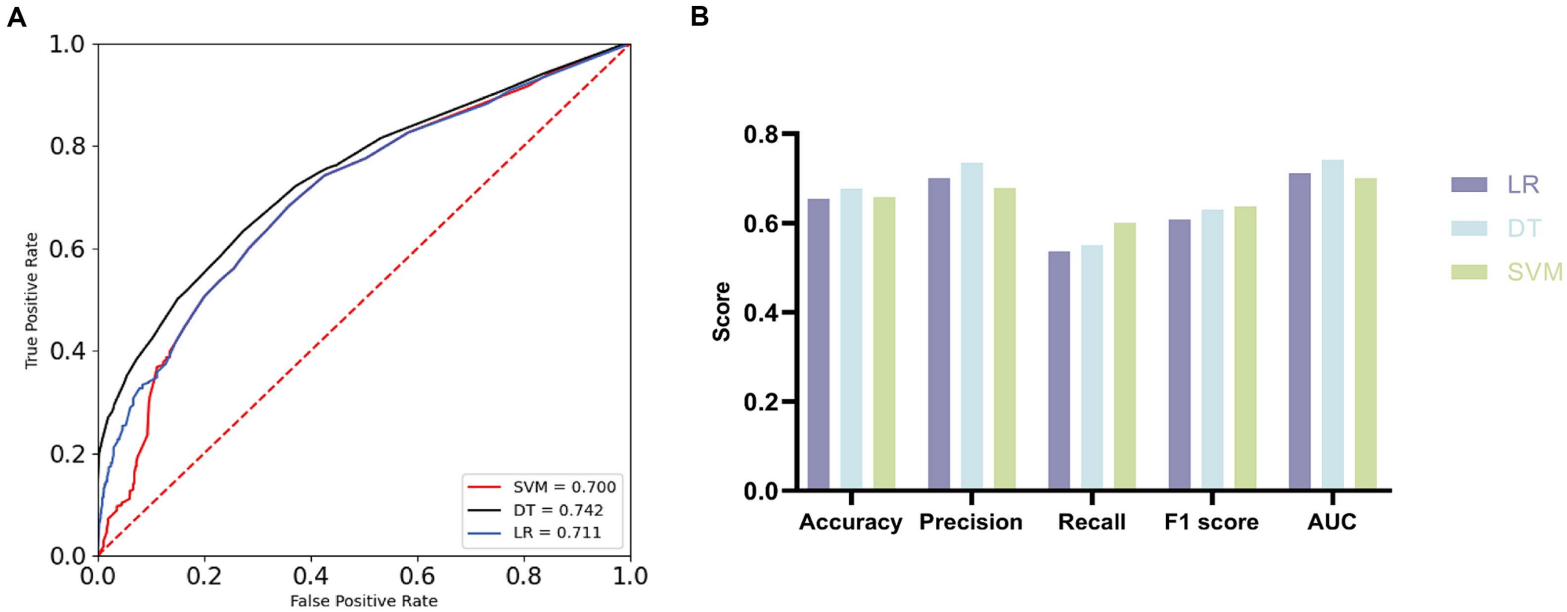
Receiver operating characteristic (ROC) curves and evaluation metrics. **(A)** ROC curves with AUC for the three machine learning algorithms. **(B)** Comparison of three machine learning algorithms.

The results (see Figure 1A) demonstrate that the Area Under the Curve (AUC) values of SVM, DT, and LR exceeded the critical threshold of 0.7, reaching 0.700, 0.742, and 0.711, respectively, confirming the diagnostic capability of these models. Comprehensive evaluation of the three machine learning algorithms using Accuracy, Precision, Recall, and F1 Score metrics (see Figure 1B; Table 7) reveals that DT exhibited outstanding Accuracy (0.677) and Precision (0.735), while SVM demonstrated superiority in Recall (0.600) and F1 Score (0.637). The combined statistical analysis and machine learning algorithm validation affirm a substantial association between ADL and EPs, indicating that impaired daily living abilities increase susceptibility to emotional problems.

**Table 7:** Evaluation of the three machine learning algorithms.

Model	Accuracy	Precision	Recall	F1 score	AUC
SVM	0.658	0.678	0.600	0.637	0.700
DT	0.677	0.735	0.551	0.630	0.742
LR	0.654	0.701	0.536	0.608	0.711

**Figure 2:**
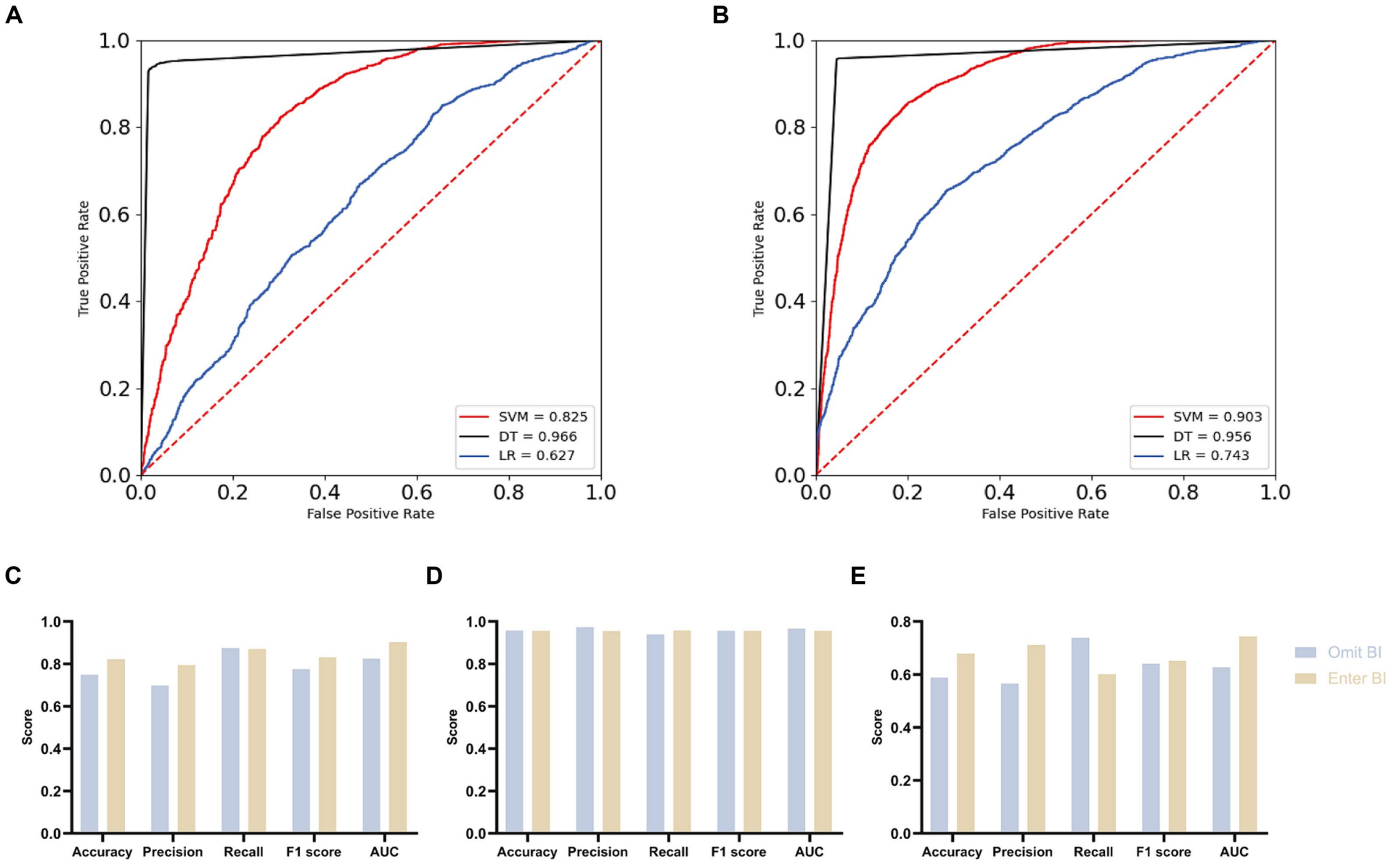
Comparison of overall effects of machine learning algorithms for two distinct datasets. **(A)** ROC curves with AUC for omit Barthel index group. **(B)** ROC curves with AUC for enter Barthel index group. **(C)** Contrasting the SVM model across the two groups. **(D)** Contrasting the DT model across the two groups. **(E)** Contrasting the LR model across the two groups.

To enhance the credibility of the association between ADL and EPs and underscore the predictive role of ADL in EPs, we partitioned the dataset into two groups with distinct features. The first group included all covariates except for the Bath Index (BI), while the second group incorporated BI while retaining the same set of covariates. Machine learning algorithms rigorously validated these two data subsets. The results demonstrate that upon inclusion of BI, the AUC values of SVM and LR models increased from 0.825 and 0.627 to 0.903 and 0.743, respectively, whereas in the DT model, the AUC value decreased from 0.966 to 0.956. Overall, the addition of BI enhanced the predictive AUC values and improved model performance (see Figures 2A,B). Further analysis using Accuracy, Precision, Recall, and F1 Score metrics (see Figures 2C–E; Table 8) revealed that the SVM model exhibited the best performance, with Accuracy increasing from 0.749 to 0.823, Precision from 0.698 to 0.794, and F1 Score from 0.776 to 0.830, demonstrating robust overall performance.

**Table 8:** Overall evaluation of three machine learning algorithms for two datasets.

Model	Accuracy		Precision		Recall		F1 score		AUC	
	Omit BI	Enter BI	Omit BI	Enter BI	Omit BI	Enter BI	Omit BI	Enter BI	Omit BI	Enter BI
SVM	0.749	0.823	0.698	0.794	0.875	0.870	0.776	0.830	0.825	0.903
DT	0.957	0.956	0.973	0.955	0.939	0.957	0.956	0.956	0.966	0.956
LR	0.588	0.679	0.566	0.711	0.739	0.601	0.641	0.651	0.627	0.743

All three machine learning algorithms verified the association between ADL and EPs and affirmed the positive role of BI in overall prediction, enhancing model performance. The validation of machine learning algorithms confirms our finding that impaired daily living abilities increase susceptibility to EPs.

## 4 Discussion

Amidst the ongoing global aging population phenomenon (40), the potential hazards of EPs among middle-aged and older adult individuals are gradually gaining attention. EPs may arise from psychological discrepancies associated with aging and are often concomitant with certain diseases (41). The exacerbation of global aging has led to increased feelings of loneliness among the older adult, coupled with the disregard for psychological well-being and emotional stability by individuals and families, thereby fostering the proliferation of EPs (42, 43). Hence, early detection and intervention for the complex factors contributing to EPs become particularly imperative.

In this study, we commenced by conducting demographic analyses of the individuals included in the research cohort. We observed disparities in the mean BI scores between cohorts afflicted with EPs and those without EPs, with the former exhibiting lower mean BI scores compared to the latter. Subsequently, utilizing Pearson correlation analysis, we established a negative correlation between ADL and EPs. Subsequent to this, employing iterative iterations of three distinct models, we conducted stratified multivariate linear regression. Even after controlling for covariates such as age, gender, residency, education, smoking and drinking status, and chronic diseases, a significant negative correlation between ADL and EPs persisted. Furthermore, machine learning algorithms validated our findings. In experiments utilizing solely BI data, the AUC scores of all three machine learning algorithms exceeded 0.7, indicating diagnostic value. In the subsequent experiments involving two groups—one with additional covariates and another with additional covariates plus BI—the inclusion of BI resulted in an overall enhancement of the predictive performance of machine learning algorithms. Particularly noteworthy was the notable improvement observed in the predictive performance of SVM when BI was added. This underscores the positive influence of BI on the predictive capabilities of machine learning algorithms, thus corroborating our findings that ADL impairment heightens susceptibility to EPs.

This study also conducted an analysis of other covariates. In the examination of demographic characteristics and Pearson correlation, gender, smoking and drinking status, hypertension, and dyslipidemia exhibited statistically significant differences between the EPs and non-EPs groups and were all significantly positively correlated with EPs. Specifically, females were more prone to EPs, while individuals with hypertension and dyslipidemia were also more susceptible to EPs, consistent with previous research findings (44–48). However, regarding smoking and drinking status, the results of this study showed that individuals who smoked less and drank less frequently were more susceptible to EPs, contradicting previous studies. We attribute this discrepancy to the higher proportion of female individuals in this study, as generally, fewer Chinese females have habits of smoking and drinking in daily life (49, 50). Additionally, studies have reported that acetylcholine contributes to regulating brain homeostasis and shaping synaptic neuron transmission and neurotransmitter levels (51). Nicotine may improve mood and alleviate anxiety by increasing acetylcholine release and the number of nicotinic receptors (52). As for other covariates such as residence and education level in this study, their definitions cannot be simply delineated through basic demographic characteristics and Pearson correlation analysis. These factors, as potential influencers of EPs, may require joint analysis with various socio-economic factors such as offspring support, retirement pensions, family migration, childhood experiences, etc., to derive more scientifically sound conclusions (53).

This study identified a significant negative correlation between ADL and EPs, which remained stable even after adjusting for other covariates, a relationship confirmed by machine learning algorithms. Therefore, emphasizing the importance of exercise for the older adults in daily life activities is crucial. Encouraging the older adults to improve ADL in community healthcare and home care settings serves as a preventive measure against EPs and ensures a better quality of life, forming the basis for quality longevity (54, 55). In the process of disease rehabilitation, such as stroke, timely restoration of ADL in patients is conducive to their psychological well-being post-illness and enhances their confidence in recovery (56, 57).

Nevertheless, this study has limitations. Its cross-sectional design precludes longitudinal exploration, hindering causal relationship establishment between ADL and EPs. Declining ADL may signify a symptom rather than a causative factor in EPs progression, inferring only a significant negative correlation between ADL and EPs. CHARLS data, collected annually, lacks precise diagnosis timings, constraining causal relationship determination. Future longitudinal studies are vital for robust evidence. Relying on “Diagnosed with Emotional Problems by a Doctor” in CHARLS, lacking detailed emotional problem classification such as Posttraumatic Stress Disorder (PTSD) and Generalized Anxiety Disorder (GAD), is another limitation. Enhancing understanding requires comprehensive emotional health assessments. Despite significant ADL-EPs correlation, predictive utility of ADL alone is limited; exploring sleep quality, social support, socioeconomic status, and chronic stress is needed to enhance predictive accuracy. Additionally, the small proportion (2.32%) of individuals diagnosed with EPs may introduce bias. Despite utilizing Synthetic Minority Over-sampling Technique(SMOTE) to address class imbalance, future studies should explore advanced techniques like stratified sampling or ensemble learning algorithms to improve result reliability. Although we examined three machine learning algorithms, further investigation is warranted for optimal predictive model identification. Nonetheless, our study’s predictive capacity remains significant (58, 59).. Furthermore, BI, derived from self-reported scales in the CHARLS dataset, differs from clinical assessments. However, literature supports the reliability of self-reported ADL assessments, validating our approach (60–62).

## 5 Conclusion

This study employs various statistical methods to reveal a negative correlation between ADL and EPs. Furthermore, the utilization of machine learning algorithms confirms this finding, indicating that impaired ADL heightens susceptibility to EPs.

### 5.1 Summary

Emotional Problems (EPs) have become a significant challenge affecting the quality of life in middle-aged and older adult populations, garnering increasing attention in public health. Early detection of potential EPs among middle-aged and older adults is crucial. This study explores the potential of Activities of Daily Living (ADL) as predictive indicators for EPs. Using data from the 2018 China Health and Retirement Longitudinal Study (CHARLS) national baseline survey, which includes 9,766 individuals aged 45 and above, we assessed ADL using the Barthel Index (BI). Statistical analyses were conducted to investigate the correlation between ADL and EPs, followed by validation using machine learning algorithms (Support Vector Machine, Decision Tree, and Logistic Regression) to elucidate the underlying relationship between ADL and EPs.

## Data availability statement

The original contributions presented in the study are included in the article/Supplementary material, further inquiries can be directed to the corresponding authors.

## Ethics statement

The studies involving humans were approved by the National School of Development at Peking University (http://charls.pku.edu.cn). The studies were conducted in accordance with the local legislation and institutional requirements. Written informed consent for participation was not required from the participants or the participants' legal guardians/next of kin in accordance with the national legislation and institutional requirements.

## Author contributions

MG: Conceptualization, Data curation, Writing – original draft, Formal analysis, Investigation, Methodology. SX: Methodology, Software, Validation, Writing – original draft. XH: Data curation, Investigation, Supervision, Writing – original draft. JH: Methodology, Software, Validation, Writing – original draft. HY: Conceptualization, Formal analysis, Project administration, Resources, Writing – review & editing. LZ: Conceptualization, Formal analysis, Funding acquisition, Project administration, Resources, Supervision, Writing – original draft, Writing – review & editing.

## Footnotes

^1^ http://charls.pku.edu.cn
