# Supplementary material for: Decoding emotional resilience in aging: unveiling the interplay between daily functioning and emotional health
Source: Front Public Health. 2024 Apr 17;12:1391033. doi: 10.3389/fpubh.2024.1391033 (PMC11061423; doi:10.3389/fpubh.2024.1391033)
Supplement: Supplementary file 1 [file Data_Sheet_1.PDF]

```python
from sklearn.linear_model import LogisticRegression
from sklearn.metrics import precision_score, recall_score, roc_curve
import matplotlib.pyplot as plt
from sklearn.ensemble import GradientBoostingClassifier, AdaBoostClassifier
import numpy as np
from sklearn.svm import SVC
import pandas as pd
from sklearn import metrics
from sklearn.preprocessing import StandardScaler
from sklearn.ensemble import RandomForestClassifier
from sklearn.metrics import accuracy_score, roc_auc_score, f1_score, classification_report
from imblearn.over_sampling import SMOTE
from sklearn.model_selection import train_test_split
from sklearn.tree import DecisionTreeClassifier
from xgboost.sklearn import XGBClassifier
import lightgbm as lgb
from sklearn.utils import shuffle

# import warnings
# warnings.filterwarnings("ignore")
data = pd.read_excel("")
data.info()
scaler = StandardScaler()

X = data.iloc[:, :-1]
y = data.iloc[:, -1]
X = scaler.fit_transform(X)

print(X)
sm = SMOTE(random_state = 2)
X, y = sm.fit_resample(X, y)
X, y = shuffle(X, y, random_state=42)
X_train, X_test, y_train, y_test = train_test_split(X, y, test_size=0.3, random_state=42)

svm = SVC(probability=True, random_state=42)# SVM
svm.fit(X_train, y_train)
svm_y_pre = svm.predict(X_test)
svm_y_proba = svm.predict_proba(X_test)

tr = DecisionTreeClassifier(random_state=42)    # DT
tr.fit(X_train, y_train)
tr_y_pre = tr.predict(X_test)
tr_y_proba = tr.predict_proba(X_test)
```

```
lr = LogisticRegression(random_state=42)    # LR
lr.fit(X_train, y_train)
lr_y_proba = lr.predict_proba(X_test)
lr_y_pre = lr.predict(X_test)

svm_accuracy_score = accuracy_score(y_test, svm_y_pre)
svm_preci_score = precision_score(y_test, svm_y_pre)
svm_recall_score = recall_score(y_test, svm_y_pre)
svm_f1_score = f1_score(y_test, svm_y_pre)
svm_auc = roc_auc_score(y_test, svm_y_proba[:, 1])
print('svm_accuracy_score: %f,svm_preci_score: %f,svm_recall_score: %f,svm_f1_score: %f,svm_
auc: %f'
          % (svm_accuracy_score, svm_preci_score, svm_recall_score, svm_f1_score, svm_auc))
print(classification_report(y_test, svm_y_pre))

tr_score = tr.score(X_test, y_test)
tr_accuracy_score = accuracy_score(y_test, tr_y_pre)
tr_preci_score = precision_score(y_test, tr_y_pre)
tr_recall_score = recall_score(y_test, tr_y_pre)
tr_f1_score = f1_score(y_test, tr_y_pre)
tr_auc = roc_auc_score(y_test, tr_y_proba[:, 1])
print('tr_accuracy_score: %f,tr_preci_score: %f,tr_recall_score: %f,tr_f1_score: %f,tr_auc: %f'
          % (tr_accuracy_score, tr_preci_score, tr_recall_score, tr_f1_score, tr_auc))
print(classification_report(y_test, svm_y_pre))
lr_score = lr.score(X_test, y_test)
lr_accuracy_score = accuracy_score(y_test, lr_y_pre)
lr_preci_score = precision_score(y_test, lr_y_pre)
lr_recall_score = recall_score(y_test, lr_y_pre)
lr_f1_score = f1_score(y_test, lr_y_pre)
lr_auc = roc_auc_score(y_test, lr_y_proba[:, 1])
print('lr_accuracy_score: %f,lr_preci_score: %f,lr_recall_score: %f,lr_f1_score: %f,lr_auc: %f'
          % (lr_accuracy_score, lr_preci_score, lr_recall_score, lr_f1_score, lr_auc))
'lr_accuracy_score: 0.768746,lr_preci_score: 0.688312,lr_recall_score: 0.147632,lr_f1_score:
0.243119,lr_auc: 0.716681'
print(classification_report(y_test, svm_y_pre))

label1 = y_test
label2 = y_test
label3 = y_test
fpr1, tpr1, thres1 = metrics.roc_curve(label1, svm_y_proba[:, 1])
fpr2, tpr2, thres2 = metrics.roc_curve(label2, tr_y_proba[:, 1])
fpr3, tpr3, thres3 = metrics.roc_curve(label3, lr_y_proba[:, 1])

roc_auc1 = metrics.auc(fpr1, tpr1)
```

```
roc_auc2 = metrics.auc(fpr2, tpr2)
roc_auc3 = metrics.auc(fpr3, tpr3)

plt.figure(figsize=(6, 6))
plt.plot(fpr1, tpr1, label='SVM = %0.3f' % roc_auc1, color='Red')
plt.plot(fpr2, tpr2, label='DT = %0.3f' % roc_auc2, color='k')
plt.plot(fpr3, tpr3, label='LR = %0.3f' % roc_auc3, color='RoyalBlue')
plt.legend(loc='lower right')
plt.plot([0, 1], [0, 1], 'r--')
plt.xlim([0, 1])
plt.ylim([0, 1])
plt.ylabel('True Positive Rate')
plt.xlabel('False Positive Rate')
plt.tick_params(labelsize=15)
plt.savefig('')
plt.show()
```
